# Force and torque on spherical particles in micro-channel flows using computational fluid dynamics

**DOI:** 10.1098/rsos.160298

**Published:** 2016-07-27

**Authors:** Jin Suo, Erin E. Edwards, Ananyaveena Anilkumar, Todd Sulchek, Don P. Giddens, Susan N. Thomas

**Affiliations:** 1 Wallace H. Coulter Department of Biomedical Engineering, Georgia Institute of Technology and Emory University, Atlanta, GA, USA; 2 Parker H. Petit Institute for Bioengineering and Bioscience, Georgia Institute of Technology, Atlanta, GA, USA; 3 George W. Woodruff School of Mechanical Engineering, Georgia Institute of Technology, Atlanta, GA, USA; 4 Daniel Guggenheim School of Aerospace Engineering, Georgia Institute of Technology, Atlanta, GA, USA

**Keywords:** computational fluid dynamics, hemodynamic force, cell adhesion, microfluidic

## Abstract

To delineate the influence of hemodynamic force on cell adhesion processes, model *in vitro* fluidic assays that mimic physiological conditions are commonly employed. Herein, we offer a framework for solution of the three-dimensional Navier–Stokes equations using computational fluid dynamics (CFD) to estimate the forces resulting from fluid flow near a plane acting on a sphere that is either stationary or in free flow, and we compare these results to a widely used theoretical model that assumes Stokes flow with a constant shear rate. We find that while the full three-dimensional solutions using a parabolic velocity profile in CFD simulations yield similar translational velocities to those predicted by the theoretical method, the CFD approach results in approximately 50% larger rotational velocities over the wall shear stress range of 0.1–5.0 dynes cm^−2^. This leads to an approximately 25% difference in force and torque calculations between the two methods. When compared with experimental measurements of translational and rotational velocities of microspheres or cells perfused in microfluidic channels, the CFD simulations yield significantly less error. We propose that CFD modelling can provide better estimations of hemodynamic force levels acting on perfused microspheres and cells in flow fields through microfluidic devices used for cell adhesion dynamics analysis.

## 1. Introduction

Dissemination and adhesion of circulating cells to distant tissues are critical to numerous pathophysiological processes ranging from atherogenesis to immune response to cancer metastasis. The dynamics of cell motion in the circulation is regulated by complex interactions between circulating cells and those lining the vasculature and takes place in the context of hemodynamic forces that regulate intravascular cell homing. Collectively, these forces influence circulating cell distribution and interactions, leading to the initiation of rolling/tethering adhesion and eventual arrest and infiltration into the surrounding tissue bed.

Accordingly, model fluidic systems have been, and continue to be, widely employed in the study of intravascular cell homing for the elucidation of contributing biological and mechanical factors [1,2]. Microfluidic approaches have not only allowed for the identification of important molecular mediators of pathological cell homing, such as in inflammation [3–5] and metastasis [6–12], but have also enabled the study of how hemodynamic forces can affect circulating cell interactions with the vessel wall via these molecular mediators. Using such model systems, the effects of increasing shear stress or shear rate on both the transport and reaction phases of cell recruitment have been demonstrated. For instance, increases in shear rate have been shown to increase collision frequencies [13–15], and some studies have demonstrated a potential effect of shear stress on cell deformability and consequentially adhesive molecule presentation [16], emphasizing the role of hemodynamically influenced transport processes that enable close contact of an adhesion molecule on a circulating cell and its conjugate receptor on the vessel wall. Furthermore, as the encounter rate between a single receptor on a circulating cell and its corresponding ligand on the vessel wall is increased when ‘slipping’ is greatest [17], the translational and rotational velocities that result from the hemodynamic flow field may also prove critical to understanding the transport phase of cell–vessel wall interactions.

In the reaction phase, rolling and adhesion of cells in close contact with a surface are mediated by fast on-rate bonds, which require high tensile strength for their maintenance. For the molecules involved in tethering and rolling of circulating cells, the strength of these bonds increases with increasing force (‘catch bond’), up to a threshold point, after which bond strength decreases with the increasing force (‘slip bond’) [13,18]. Furthermore, a comparison between tethering behaviour of rigid polystyrene spheres functionalized with adhesive molecules and deformable cells presenting the same molecules reveals similarities that emphasize the role of force regulation of bond behaviour despite differences in cell or particle deformability and subsequent adhesive molecule presentation [15,18]. Following these initial tethering and rolling events, hemodynamic forces continue to regulate the circulating cell adhesion cascade by affecting the ability of tethered cells to firmly adhere to the endothelium and subsequently extravasate. Recent studies have demonstrated a role for the engagement of some adhesion molecules and cytokine receptors in the affinity and avidity of other adhesion molecules co-expressed on the same cell [19–21]. Importantly, these studies have suggested that force plays a crucial role in regulating the intracellular signalling processes that actuate these affinity and avidity changes with consequences in eventual cell extravasation [22,23].

In order to better understand the adhesion characteristics of cells in micro-channel flow experiments, a thorough and precise determination of the forces acting on cells and the resultant translational and rotational velocities of the cells is necessary. Numerous investigators have employed an elegant fluid dynamics model proposed almost 50 years ago by Goldman *et al.* [24] as the basis for estimating forces and torques on cells flowing near a surface. In that theoretical model, a constant shear rate, corresponding to a linear velocity profile, was assumed, and the nonlinear equations of fluid motion were simplified to a linear system (Stokes flow) valid in the limit of small Reynolds numbers. These assumptions have three limitations. First, in a fully developed channel flow, the velocity profile is parabolic and thus the shear rate is not constant, especially when the diameter of the cells cannot be ignored relative to the size of the channel section. Second, the simplifying assumptions in the Goldman model become less acceptable as the Reynolds number increases and nonlinear effects come into play. Third, cells are not stationary in most experiments so that a translating and rolling cell will interact with flow making the shear rate over the cell surface complex. Computational fluid dynamics (CFD), in which three-dimensional Navier–Stokes equations are solved numerically, combined with mesh-update methods [25] can simulate the flow field around a moving sphere so that the hydrodynamic forces that are composed of shear stress and pressure on the surface of the sphere can be more accurately computed, leading to better predictions of particle translation and rolling near surfaces.

In this study, we develop a computational framework for solution of the three-dimensional Navier–Stokes equations focusing on sphere sizes and flow conditions that are representative of experimental investigations of cell dynamics in microfluidic flows, and we compare the full three-dimensional solutions with the results calculated from the Goldman model. Additionally, we have performed a series of experiments in a microfluidic device to measure the translational and rotational velocities of inert spherical particles over a range of shear rates relevant to cell dynamics studies, and we compare the two theoretical approaches with experimental data. While both the computational and experimental work reported here are at very low Reynolds numbers and hence the convective terms in the Navier–Stokes equations are small, the approach is valid for situations at higher Reynolds numbers.

## 2. Material and methods

### 2.1. Computational approach

**Figure 1.**
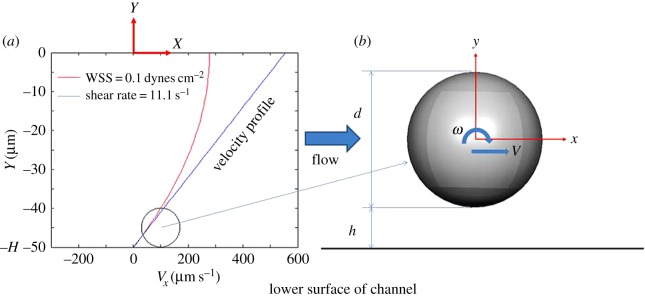
(*a*) A parabolic velocity profile (red curve) and the linear velocity profile (blue line) both produce the same wall shear rate and wall shear stress (WSS) on the lower surface of the channel. The channel height is 100 µm and the sphere diameter is 10 µm. The figure depicts the case for WSS = 0.1 dynes cm^−2^ corresponding to a wall shear rate of 11.1 s^−1^. (*b*) The sphere translates with velocity *V* along the *x*-axis and rotates with angular velocity *ω* about the *z*-axis. The gap between the lower channel surface and the sphere surface is *h*.

We employed dimensions for the sphere diameter and microfluidic channel that are representative of a set of experiments performed in our laboratory (described subsequently). While the spherical particle shape is a limitation to the direct physiological application of these calculations to non-spherical cells, the convention of the commonly used Goldman model as well as our experimental system dictates that such an assumption is appropriate to enable comparisons between these situations and our results. The configuration of the system is described in figure 1. Two Cartesian coordinate systems are used, the flow system *XYZ* (figure 1*a*) and the particle system *xyz* (figure 1*b*). The origin of the *XYZ* system is located at the centre point of the channel, while the *xyz* system has its origin fixed at the centre of the sphere. The *X*-axis is taken to be in the flow direction and the *Y*-axis is perpendicular to the lower surface of the channel. In micro-channel flows, the Reynolds numbers are sufficiently low that laminar, incompressible conditions prevail leading to the following form for the three-dimensional Navier–Stokes and continuity equations:

2.1*a* $$\frac{\partial \text{V}}{\partial t}+\text{V}\cdot \nabla \text{V}=-\frac{\nabla P}{\rho }+\nu \nabla^{2}\text{V}$$

and

2.1*b* ∇⋅V=0, 

where the velocity $\text{V}=V_{x}\text{i}+V_{y}\text{j}+V_{z}\text{k}$, *ρ* is the fluid density, *ν* is the kinetic viscosity and *P* is the pressure. The channel height in our study is set at 2 × *H* = 100 µm.

Goldman calculated two sets of results corresponding to two conditions, one in which the sphere was stationary and one in which the sphere was in free motion [24], so our CFD computations were also restricted to these two conditions. When the sphere is stationary, the first term of equation (2.1*a*) can be ignored because flow is steady. In the free motion state, we assume that the translational velocity and angular velocity of the sphere are constant and that the summation of forces acting on the sphere is zero. In this investigation, the effects of gravity are neglected.

**Figure 2.**
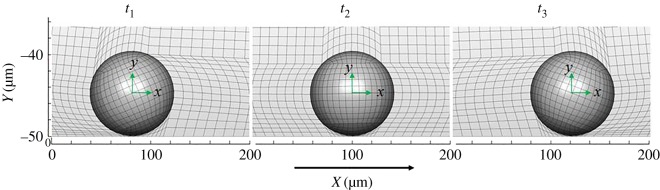
The numerical mesh is placed at fixed locations on the sphere's surface and rotates with the sphere as it translates in the *X* direction. The origin of the (*x*, *y*, *z*) coordinate system is located at the centre of the sphere and translates with its motion. Numerical computations adjust for the moving mesh at each stage, e.g. convergence is obtained at a given time step and the sphere translates and rotates under the actions of net force and torque. Steady state translation and rotation are achieved within 20 computational time steps.

In the case of free motion, the numerical mesh must account for both translation and rotation of the sphere. We chose to fix the mesh on the sphere surface and continuously updated the mesh near the sphere to match the cell motion, as shown in figure 2. The main influence of mesh-updating for numerically solving the Navier–Stokes equations is that the convective term (the second term of the left-hand side of equation (2.1*a*)) must account for the mesh motion. Briefly, we input an initial estimate of the translational and rotational velocities of the sphere into the simulation, and the mesh was then repeatedly refined according to the current position of the sphere, which is a function of the velocities and a small time step. A subprogram for mesh calculation was called automatically when the mesh needed to be refined, and force and torque were obtained at that step. From these, new velocities were input until the force and torque approached zero at that transient step, and the translational and angular velocities were determined.

Goldman's model assumed flow over the sphere with a constant shear rate that did not consider the geometric sizes of the flow channel; however, a CFD simulation considers these sizes and conditions on the boundaries. Characteristics of micro-channels are that the channel length is much larger than its width and the width is much larger than the height (14 × 2 × 0.1 mm in our experiment). A very fine numerical mesh is required to resolve flow in the neighbourhood of the sphere, yet meshing the entire channel at the same spatial resolution is computationally prohibitive. Thus, we employed multi-scale meshing and a reduced domain for computations in a manner that did not sacrifice accuracy in the region of interest. In restricting our computational domain, the two side and upper boundaries of the domain are assumed to follow an inviscid relationship between velocity and pressure in the CFD simulation. Based on these conditions, the inflow to the domain is modelled as steady flow between parallel plates with slip velocity along the *X* direction at the upper boundary, and the flow through inlet section in the CFD domain can be rewritten from equation (2.1*a*) as

2.2 $$\frac{\partial^{2}V_{x}}{\partial Y^{2}}=\frac{1}{\mu }\frac{\partial P}{\partial X},\;$$

where *µ* is the dynamic viscosity of fluid.

The wall shear stress (WSS) is defined by

$$\mathrm{WSS}={\left|\mu \frac{\partial V_{x}}{\partial Y}\right|}_{Y=-H}$$

and substituting this definition into equation (2.2) gives the expected parabolic velocity distribution in the inlet section of the reduced computational domain as

2.3 $$V_{x}=-\frac{1}{2\mu H}(Y^{2}-H^{2})\cdot \mathrm{WSS},\;$$

where −H≤Y≤H.

Because the inlet velocity distribution is also a function of *H*, the CFD modelling is individual for different sizes of channels. In our case, a parabolic profile with height = 100 µm is shown in figure 1*a* where the WSS on the lower surface is 0.1 dynes cm^−2^. We varied the WSS parameter in the computations but kept the height fixed in this study because this was the configuration used in our *in vitro* experiments employed for validation.

In order to obtain good spatial resolution in the neighbourhood of the sphere and yet avoid an unmanageable mesh size, the width and height of the computational domain were prescribed to be 80 µm and 40 µm, respectively. Under this condition, the diameter of the sphere (10 µm) is no longer negligibly small with respect to the computational domain's cross section. To compensate for this effect on the flow field, the side and upper boundaries in our CFD domain were smoothly enlarged from the inlet section, reached a maximum value over the sphere, and then were symmetrically decreased to the original dimensions at the outlet section in such a way that the cross-sectional area available to the flow remained the same. This allowed us to enforce inviscid boundary conditions and thus avoid requiring outflow through these surfaces. To ensure the accuracy of this approach, computations were performed for a case in which the domain dimensions were doubled, and we found that the effects on the computed flow field were negligible.

The reduced computational domain is shown in figure 1*a*. The sphere, located near the lower surface, is shown in figure 1*b*, and the origin of the *xyz* system is located at the centre of the sphere. The computational *x*-axis is taken to be along the flow direction (channel *X*-axis), and the *y*-axis and *z*-axis are parallel to the channel *Y*-axis and *Z*-axis. The sphere of diameter *d* translates in the *x* direction and rotates about the *z*-axis, so that *u* and *ω* are its translational and angular velocities. The gap between the lower surface and the sphere surface is *h*.

The total force exerted on the sphere is calculated by integrating the hydrodynamic forces acting on the surface: the viscous shear stress and pressure both produce a net force to push the sphere in translation but only the former produces a torque to cause rotation. The integration of force includes every cell of the mesh around the sphere's surface

2.4 $$\text{F}=\sum_{i=1}^{N}({\text{P}}_{i}{\left(\Delta s\right)}_{i}+\tau_{i}\cdot {\left(\text{n}\Delta s\right)}_{i}).$$

The integration of torque is similar to that for the force but must consider the radius vector of each cell of the mesh

2.5 $$\text{T}=\sum_{i=1}^{N}{\text{r}}_{i}\times (\tau_{i}\cdot {\left(\text{n}\Delta s\right)}_{i}).$$

Here, ***P***_*i*_ is the pressure and *τ_i_* is the viscous shear stress on the *i*th cell of mesh, ***r***_*i*_ is the radius of the cell in *xyz* system and Δ*s_i_* is the area of the cell. *N* is the number of cells on the sphere's surface.

Solutions to the Navier–Stokes equations were performed with the CFD-ACE+ commercial finite volume code (ESI Group, Paris) on a desktop computer. The simulations were performed for three successively finer meshes. For the fewest number of mesh points (lowest spatial resolution), the computations gave values for hydrodynamic force Fx = 6.01 pN and torque Tz = 10.71 pN µm for the case of WSS = 0.1 dynes cm^−2^. Next, the number of mesh points was doubled in each spatial dimension and computations resulted in values of Fx = 6.64 pN and Tz = 11.51 pN µm. Finally, when the number of mesh points was again doubled in each spatial dimension, we computed Fx = 6.65 pN and Tz = 11.52 pN µm, so that Fx increased 0.1% and Tz increased 0.08%. Thus, we concluded that we had achieved independence between the latter two mesh sizes. For the stationary sphere, the computational time was of the order of 1 h to achieve convergence. For the freely moving sphere, small time steps were employed so that the iterations required to reconfigure the moving mesh at each step converged and thus established the new position of the sphere before the next time step. The number of steps required to reach steady translational and rotational velocities varied, depending on the input parameters, but this state was typically achieved within approximately 20 time steps. Consequently, computational time was of the order of 20 h for each case using our desktop computer.

### 2.2. Experimental methods

#### 2.2.1. Materials

The polydimethylsiloxane (PDMS) base and curing agent were from Ellsworth Adhesives (Germantown, WI, USA). Glass slides (2^″^ × 3^″^) were purchased from VWR (Randor, PA, USA). Non-tissue culture treated polystyrene plates (245 × 245 mm) were purchased from Corning (Corning, NY, USA), and 10 µm yellow-green fluorescent polystyrene microspheres were obtained from ThermoFisher Scientific (Waltham, MA, USA).

#### 2.2.2. Perfusion chamber fabrication

An aluminium block (Alloy 6013) mould with a negative feature for the PDMS chamber was fabricated using a micro-milling machine (OM 1-A, HAAS, Oxnard, CA, USA). PDMS was prepared at a ratio of 9 : 1 base to curing agent, poured into the mould and cured for 3 h at 90°C. The resultant PDMS block contained an open 2 mm wide by 0.1 mm deep channel that formed a closed channel with a planar surface. A 0.1 mm deep settling feature upstream of this channel consisted of a 10.9 mm linear inlet region followed by a transition into a circular channel with inner and outer radii of 10.5 and 11.75 mm, respectively. This specific design has been characterized previously and applied to cell rolling studies [26]. The PDMS channel inlet and outlet were created using a biopsy punch, and the channel was mounted to glass slides spin-coated (WS-400BZ-6NPP-LITE, Laurell, North Wales, PA, USA) with a 10 : 1 base to curing agent PDMS mixture and treated at 50°C overnight for completion of chamber fabrication.

#### 2.2.3. Janus particles

Yellow-green fluorescent polystyrene microspheres (10 µm; ThermoFisher Scientific, Waltham, MA, USA) were coated with gold to an approximate thickness of 0.15 µm using a metal evaporation process previously described [27]. The resulting Janus particles were suspended at a concentration of approximately 5 × 10^5^ particles ml^−1^ in 0.1% bovine serum albumin (BSA) in Dulbecco's phosphate-buffered saline (D-PBS) with calcium and magnesium.

#### 2.2.4. Perfusion experiments

**Figure 3.**
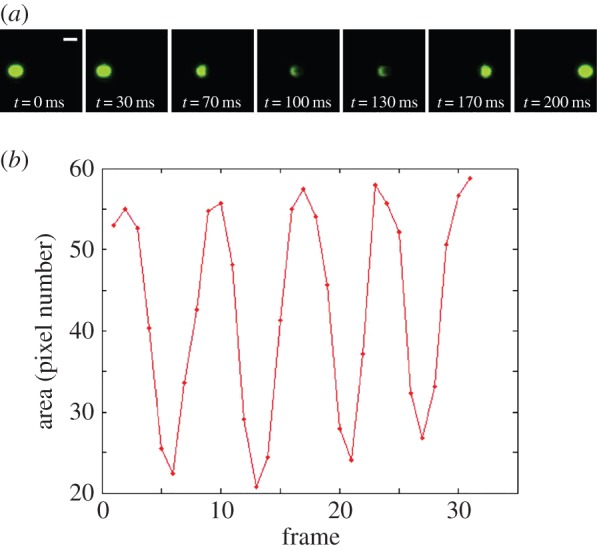
(*a*) Interleaved videos were acquired using NIS-Elements (Nikon) with identical camera and software settings for each experiment. A series of frames of the same particle is shown on one image (frame rate = 25 frames s^−1^). Panel (*b*) presents the area of each contour of the particle at different times (frames).

Prior to use, perfusion chambers were blocked with 1% BSA in D-PBS with calcium and magnesium for 1–2 h at room temperature. Next, fittings enabled the connection of tubing in line with the device, which led to a reservoir on the inlet end and a syringe on a syringe pump on the outlet end. The tubing and chamber were filled with 0.1% BSA in D-PBS with calcium and magnesium. The chamber was placed on an optical microscope (Eclipse Ti, Nikon, Melville, NY, USA) and the focal plane was set approximately 5 µm above the bottom of the chamber. The syringe pump was set to the withdraw mode, and the desired WSS was established by controlling the flow rate. After reaching steady state, a pulse of Janus particle suspension was added to the inlet reservoir, and 20 min movies were imaged using a fluorescein isothiocyanate filter (excitation 475–492, emission 505–535; Chroma, Bellows Falls, VT, USA) approximately 5 mm from the channel outlet. Interleaved videos were acquired using NIS-Elements (Nikon), with identical camera and software settings for each experiment (figure 3*a*). For all experiments, the exposure time was 0.281 µs, the frame rate was 25 frames per second, the objective magnification was 10×, the image size was 960 by 500 pixels and the image was binned 2 × 2.

#### 2.2.5. Video analysis

Five-minute videos of particle perfusion experiments performed at prescribed chamber locations were analysed using a custom particle tracking software as previously described [26]. An image series of a particle is shown in figure 3*a*. The contrast was first applied to detect fluorescent Janus particle edges and the X and Y positions of the centroids of each detected particle were stored in the first frame. Particle position and size were subsequently recorded until leaving the field of view and the particle could no longer be detected. Tracking was constrained by considering forward less than half of the pixels of the previously detected particle diameter, calculated as the radius of the minimum contour-enclosing circle, and a divergence angle of 15° to the flow direction. Mean particle velocity, based on the total tracked distance and video frames per second, was calculated; and the frame-resolved projected area of each bead was recorded. Videos were analysed by modifying the OpenCV-based Traffic Flow Analyzer (https://github.com/telescope7/TrafficFlowAnalysis) as previously described. The contour detection algorithm defines particles as more than 5 µm in diameter that are stored in the Moving Object database and compared to other previously tracked objects and mapped to the appropriate moving instance based on forward movement (using either a look ahead window or an overlapping object boundary analysis). Once the object was no longer tracked or exited the field of view, the object data were read to file. The areas of each detected contour (particle) were different because the particles were in different rotational positions. The continuous change of detected area provided the rotational position of the particle, and one cycle of the change represented a revolution of the particle as figure 3*b* shows. The mean angular velocity of particles was calculated based on the number of cycles and frame rate.

## 3. Results

### 3.1. Stationary sphere computations

**Figure 4.**
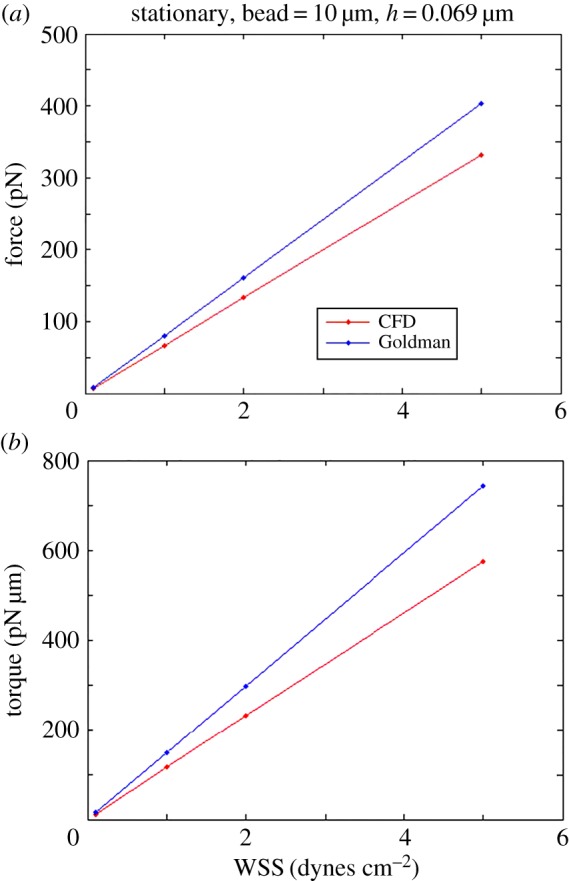
(*a*) The relationship between hydrodynamic force (along the *x*-axis) and WSS when the sphere is stationary and the gap height is 0.069 µm. The results of the Goldman model are consistently larger than those computed using the CFD approach that assumes a parabolic velocity profile, and both values increase as the WSS increases. (*b*) The relationship of hydrodynamic torque (around the *z*-axis) and WSS under the same conditions as in (*a*).

The force and torque acting on the sphere were computed for several cases in order to compare with predictions from the Goldman model. We considered a sphere of 10 µm in diameter and varied the shear rate and gap height. Table 1 summarizes the results for a channel inlet shear rate = 11.1 s^−1^ that induces WSS = 0.1 dyne cm^−2^ on the lower surface when the fluid viscosity is *µ* = 0.0009 N s m^−2^. The gap between surface and sphere is taken as *h* = 0.069 µm. The computed hydrodynamic force and torque on the sphere are 8.31 pN and 14.51 pN µm using the CFD simulation and assuming a linear velocity profile at the inlet to the computational domain. When employing a parabolic velocity profile that would be characteristic of flow in a microfluidic channel, the CFD simulation gives a hydrodynamic force and torque on the sphere of 6.64 pN and 11.51 pN µm, respectively. According to the Goldman model, the computed force and torque are 8.07 pN and 14.83 pN µm for these shear rate conditions. The results obtained by the CFD simulation with a linear profile are in good agreement with the Goldman model, giving assurance that the numerical approach is valid, as expected for these low Reynolds numbers. The deviation in results using Goldman's linear profile assumption rather than a parabolic profile as modelled using CFD is approximately 25% for both force and torque, and this ratio is relatively constant over a wide range of WSS values (figure 4). Importantly, the difference between results for the parabolic inlet profile and the linear profile will increase as the ratio of the channel height to the sphere diameter decreases.

**Table 1.** Computed force and torque on a stationary sphere with diameter = 10 µm and gap height *h* = 0.069 µm. Flow conditions were set to give a wall shear rate of 11.1 s^−1^ corresponding to WSS = 0.1 dynes cm^−2^. Case A represents a fully three-dimensional CFD solution; Case B employs full CFD but with the inlet flow situation being linear, as assumed by Goldman; and Case C is the solution obtained using the Goldman model. The differences between linear and parabolic profiles are approximately 25% for both force and torque. The good agreement between Cases B and C serves as a validation of the CFD approach.

	Case A. Full CFD with parabolic profile at WSS = 0.1 dynes cm^−2^	Case B. Full CFD with linear profile at shear rate = 11.1 s^−1^	Case C. Goldman solution for linear profile at shear rate = 11.1 s^−1^
force (pN)	6.64	8.31	8.07
torque (pN µm)	11.51	14.51	14.83

**Figure 5.**
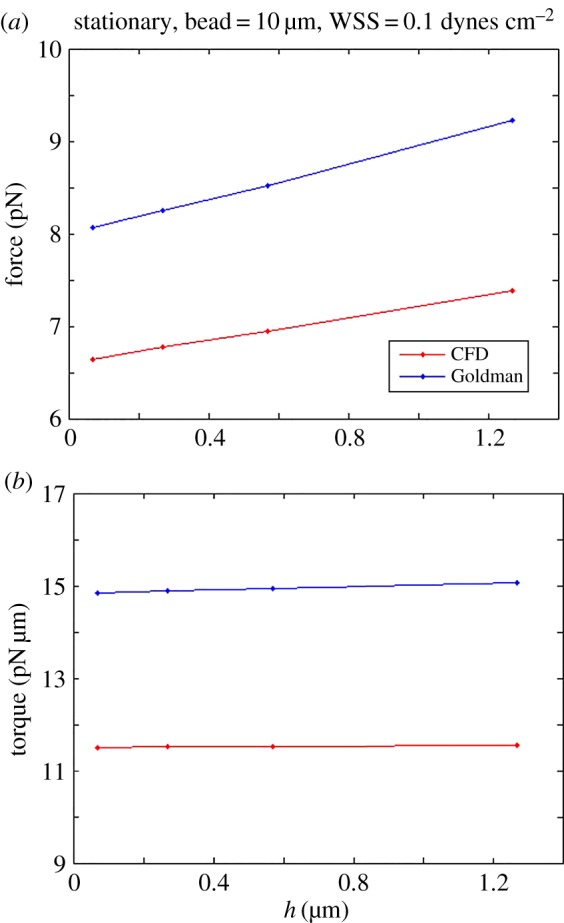
(*a*) The relationship between hydrodynamic force (along the *x*-axis) and gap height *h* when the sphere is stationary and WSS = 0.1 dynes cm^−2^. The force calculated from the Goldman model is larger than for the CFD approach that assumes a parabolic velocity profile, and both values increase as the gap height increases. (*b*) The relationship between hydrodynamic torque (about the *z*-axis) and gap height under the same conditions as in (*a*). Again, the Goldman model results are consistently larger, although both sets of torque values are fairly constant as the gap height changes over the range investigated.

When other conditions are fixed, the force and torque change with different gaps as shown in figure 5. Results from the CFD model when assuming a parabolic velocity profile are always lower than those of Goldman over the range of gaps we investigated. This arises largely from the fact that the Goldman model enforces a linear velocity for all gap heights, exacerbating the deviation from a parabolic profile that would be expected in actual microfluidic experiments. The force and torque vary almost linearly with the gap height, although the force increases more quickly as the gap increases, while the torque variation is much smaller.

### 3.2. Freely moving sphere: experiments and computational fluid dynamics

**Figure 6.**
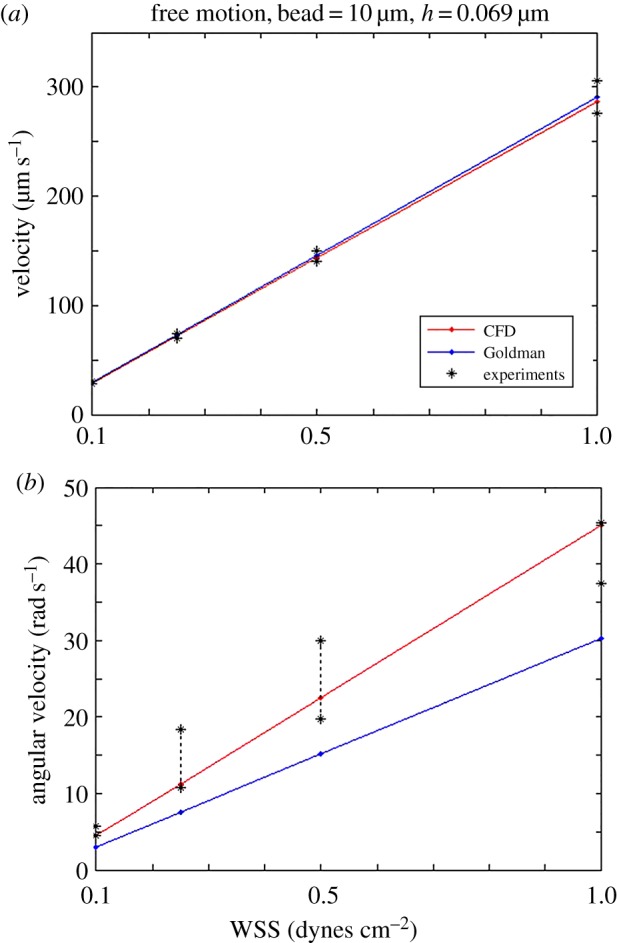
(*a*) The relationship between translation velocity of a freely moving sphere and WSS. The results of Goldman model, CFD approach that assumes a parabolic velocity profile, and experiment show remarkable similarity. (*b*) The relationship of angular velocity of the sphere in free motion and WSS. The CFD results are in good agreement with experiment, but the Goldman model significantly underestimates the experimental results.

Our experimental methods captured both the translational and rotational velocities for fluorescent Janus particles. These were measured from the translational movement of individual particle fluorescence through the imaging field of view and by analysing the periodicity of the change in visible particle fluorescence at WSS levels of 0.1–1.0 dynes cm^−2^. We then compared these results to our simulations of a freely moving sphere at shear rates corresponding to WSS ≤ 1.0 dynes cm^−2^. Figure 6*a* presents computed and measured translational velocities when WSS = 0.1, 0.25, 0.5 and 1.0 dynes cm^−2^. The standard deviation around the mean of the measured individual sphere velocity values is shown as four pairs of black stars in figure 6*a*, indicating that 68% of the velocity values of the experiment are located within that range at that WSS level. As figure 6*a* shows, the translational velocities of spheres determined from the CFD results, the Goldman results, and the experimental results are in good agreement.

A similar process was used for determining the rotational velocity of individual spheres in the experiments, and the results are shown in figure 6*b*. The experimental results are presented as four individual data distributions corresponding to four values of WSS. The rotational velocity shows a linear relationship with WSS for both CFD results as well as for the Goldman model, but the values computed from the latter are significantly lower. For example, at WSS = 0.1 dynes cm^−2^, the rotational velocity is predicted to be 5.0 rad s^−1^ using the CFD method but only 3.1 rad s^−1^ using the Goldman method. Although there is some scatter in the experimental data, the agreement between theory and experiment is clearly superior for the CFD methodology.

We compared the CFD and Goldman models with another set of experimental data [28] in which individual cells of a lymphoid cell line origin (with average diameters of 12 µm) were perfused through a parallel plate flow chamber at a shear rate of 1.32 s^−1^ and their translational and rotational velocities recorded by tracing changes in the positions of cell surface patterns. The CFD approach was again superior to the Goldman model insofar as agreement with experimental data is concerned (table 2).

**Table 2.** Comparison of CFD and Goldman models with experimental results from Tissot [28]. Experimental conditions were: fluid viscosity *μ* = 0.001 N s m^−2^; fluid density *ρ* = 1000 kg m^−3^; shear rate = 1.32 s^−1^; cell diameter = 12 µm; gap height = 1.4 µm. Both the CFD and Goldman models are in agreement with the experimentally obtained translational velocity. However, the Goldman model underestimates the measured rotational velocity by approximately 31%, while the CFD result is in good agreement with experimentally obtained results.

	translational velocity (μm s^−1^)	angular velocity (rad s^−1^)
Tissot's experiments	7.07	0.78
CFD results	7.60	0.81
Goldman model	7.88	0.54

### 3.3. Free motion at higher wall shear stress

Cell dynamics studies in microfluidic flow chambers have been reported over venous WSS values that range from 1 to 5 dynes cm^−2^ [1]. Consequently, we extended the range of our computations for WSS values up to 5 dynes cm^−2^ and compared with the Goldman model. The translational velocities computed by both methods were found to be in good agreement, while the ratio of rotational velocities from the CFD and Goldman model computations is consistently equal to 1.49 over this range of WSS conditions.

## 4. Discussion

Numerous investigators have interpreted results from cell dynamics experiments employing the Goldman theoretical model for Couette flow over a sphere near a single surface at very low Reynolds numbers, i.e. Stokes flow. By relating various measurements of translational and rotational velocity to parameters in the Goldman model, it was possible to estimate parameters such as shear rates in vessels [29], define rolling cells versus those in the free fluid stream in videomicroscopy analysis [30], calculate bond encounter rate versus duration for cells in contact with adhesive substrates in shear flow [17] and to model transitions between cell adhesive states [31]. In this study, we investigate the consequences of removing certain limitations in the Goldman model using numerical techniques; specifically, we employ the full three-dimensional Navier–Stokes equations and a parabolic near wall velocity profile to compute translational and rotational velocities for neutrally buoyant spheres under conditions representative of cell dynamics studies in microfluidic channels, and we compare our predictions with those using the Goldman model. We computed force and torque on a stationary sphere, followed by computations for translational and rotational velocities for freely moving spheres of 10 µm in diameter over a range of wall shear rates corresponding to WSS values from 0.1 to 5.0 dynes cm^−2^, values often used in cell dynamics studies.

We observed that computed force, torque, as well as translational and rotational velocity varied linearly over the range of parameters investigated, regardless of whether the equations were Stokes flow (Goldman) or the full Navier–Stokes equations (current computations). Although at higher Reynolds numbers nonlinearities would undoubtedly come into play, our study suggests that a limitation of the Goldman model for many cell dynamics studies is the assumption of a linear velocity profile rather than the neglect of the convective terms in the Navier–Stokes equations. This velocity profile assumption emphasizes the importance of the ratio of sphere diameter to channel height as a factor in experimental investigations.

On the other hand, while trends were similar between the models, there were notable differences in computed values. For the stationary 10 µm sphere, the force and torque computed from the Goldman model were approximately 25% greater than predicted by the CFD model at WSS = 0.1 dynes cm^−2^ for all gap heights studied (figure 5). This suggests that, even at these low shear stress conditions, the Goldman model can result in significant errors in the estimated bond strength required for cell attachment. For the freely moving sphere, the translational velocity computations from the two models agree well, but the rotational velocities are notably different (figure 6). Rotational velocity will affect the rate at which cell receptors are presented to surface ligands, and thus errors in estimating this rotation may misinform the rate as well as extent of bond formation in experimental studies.

The differences between the two models have implications for the computed translational and rotational velocity values. While the force and torque computed by the Goldman approach might be ‘adjusted’ by a multiplier using the CFD result, there is a difference in how the translation and rotation are computed. In the Goldman approach, the interaction effects of the rotating sphere upon flow in the immediate sphere neighbourhood are not precisely modelled, whereas in the CFD method these induced velocities are accounted for by the moving mesh approach. This difference can be seen in figure 6 where the translational and angular velocities are plotted as a function of WSS. Note that although the force and torque on the sphere as computed by the Goldman model are approximately 25% greater than for the CFD model, the two predictions for translational velocities are virtually identical and the CFD model predictions for rotational velocity are approximately 50% larger than those for the Goldman model over the range 0.1 < WSS < 5.0 dynes cm^−2^.

As a test of the theoretical methods, we compared these computational predictions with experiments in our laboratory (figure 6) and with those reported by Tissot [28], shown in table 2. Our experiments were limited to WSS ≤ 1.0 dynes cm^−2^, but it is seen that over this range the agreement between theory and experiment for rotational velocity is much better using the CFD approach. The same can be said for agreement with the Tissot data (table 2). These results suggest that the neglect of the interaction of the moving sphere with the flow resulting from the linearized (Stokes) approach compensates to a degree for the overestimation of force so that the translational velocity is approximately in agreement with the CFD model and with experiment. On the other hand, the overestimation of torque by the Goldman model fails to produce rotational velocities predicted by the CFD model or those obtained experimentally.

This study has implications for interpreting experimental data from cell dynamics experiments in microfluidic channels. For example, numerous investigations have estimated the force acting on adherent cells in flow [32–34] and elaborated on the force sensitivity of the off rate of receptor–ligand bonding [35–38]. Others have utilized translational velocities to estimate the distance of cells in the free fluid stream from the wall [32] or the force acting on surface-bound cells [35] to investigate membrane tether formation between neutrophils and spread platelets or calculate spring constants of cell microvilli, respectively. Our studies suggest that using the Goldman model for estimating force and torque can lead to errors of the order of 25% or more, depending upon the dimensions of the microfluidic devices and cell sizes. In particular, the use of microfluidic devices comprised of increasingly shallow channel heights for experiments investigating cell adhesion processes, which have increased with the widespread utilization of advanced microfabrication techniques, will result in even higher levels of error in estimated levels of hemodynamic force due to the significantly decreased ratio of channel height to the diameters of perfused cells.

While we do not study any adhesive bonding behaviour directly in these simulations or experiments, as inert Janus particles are perfused through passivated microfluidic channels, a precise determination and experimental verification of force and torque on non-adhering cells are critical to understanding how adhesive bonds dynamically respond to different levels of such forces. Not only is it expected that different adhesive receptor–ligand bonds may have different static properties, but the dynamic behaviour of these bonds depends on the forces acting on the bonds. This is exemplified in the commonly observed catch versus slip bond behaviour of a single adhesive receptor–ligand pair [13,18]. From a clinical standpoint, understanding these adhesive bond dynamics is critical to informing the development of therapeutics for selective interference of pathologically relevant cell adhesion, such as metastatic cell dissemination. As adhesive bond dynamics of various types of adhesive receptor–ligand bonds may be differentially regulated by force and torque, errors of the order of 25% in force and torque estimations could significantly misinform the quality and extent of bonding that occurs during pathology. Such errors thus have potential ramifications in the development of effective therapeutic interventions aiming to interfere with specific adhesive receptor–ligand pairs, as well as dosing schemes for the selective interference of pathological but not physiological cell adhesion and homing. Our findings suggest that CFD approaches such as those described in this paper could therefore aid in the more precise determination of adhesion bonding mechanisms relevant to cancer metastasis and inflammation.

A drawback of the CFD model is its complexity. Each case must be computed individually, and the computations are time consuming. On the other hand, the Goldman model is amenable to using tabulated values for the desired variable, so that the computations become arithmetic and thus simple. Because the behaviour of desired variables—force, torque, translational and rotational velocity—is linear over a range of conditions of interest in cell dynamics studies, it may be useful to explore the suitability of simple ‘corrections’ determined from a limited number of CFD scenarios that could be applied to the tables provided by Goldman *et al.* [24]. These ‘corrections’ would, however, be dependent upon the dimensions of the microfluidic channel employed and would require computation of numerous situations in order to develop an empirical correction. Other computational approaches apart from solving the Navier–Stokes with CFD employed here can be taken to simulate cells in hydrodynamic flow. In particular, lattice Boltzmann method [39–41], multiparticle collision dynamics [42] and dissipative particle dynamics [43] enable consideration of lift forces arising from large Reynolds numbers or cell deformability. These methods also allow the simulation of large systems and can therefore be used to determine the forces and torques acting between a deformable cell and its substrate as well as the more challenging case of multiple spheres or cells not studied here. By including a variety of physical effects, these techniques allow for accurate prediction, but are also computationally intensive and can only simulate short distances/time scales. In any case, investigators should balance the level of accuracy needed in deriving cell dynamics parameters from experimental data versus complexity of the theoretical approach.

The major significance of the study is related to contributing to the improved elucidation of force-regulated cell adhesion dynamics using controlled *in vitro* experimentation. Such information could then be transferred to fluid dynamic investigations that are specific to various clinical *in vivo* applications, e.g. monocyte adhesion to vascular adhesion molecules expressed on endothelium in the case of atherosclerosis or cancer metastasis. At low Reynolds numbers and when the dimension for vessel curvature is large with respect to vessel diameter, flow streamlines will be parallel to the vessel surface. Hence, as long as cell diameter is small with respect to vessel diameter, the model presented could be applied directly to predict cell adhesion. Other situations, such as atherogenic processes at arterial branches, would require altering the hemodynamic model to be more specific to the case at hand. Cell attachment would then be dependent on the bond strengths (determined from *in vitro* studies) and the local hemodynamic forces.

## 5. Conclusion

We developed a computational framework for solution of the three-dimensional Navier–Stokes equations using CFD focusing on sphere sizes and flow conditions that are representative of experimental investigations of cell dynamics in microfluidic flows. We find that the full three-dimensional solutions that use a parabolic velocity profile more closely match the measured translational and rotational velocities of inert spherical particles perfused through a microfluidic device over a range of WSS levels relevant to cell dynamics studies than those predicted by CFD and the Goldman model that assume a linear velocity profile. Accordingly, estimations of particle translation and rolling behaviour near surfaces when using the Goldman model can result in errors, the magnitude of which can vary significantly depending on experimental conditions. Though complex and time consuming, CFD modelling to simulate the flow field around a moving sphere can result in better estimations of the levels of hemodynamic force acting on perfused microspheres and/or cells in flow fields through microfluidic devices used for cell adhesion dynamics analysis.

## Supplementary Material

Files included contain raw data of particle traces used in the analysis.

## Supplementary Material

Raw data

## Data accessibility

The datasets supporting this article have been uploaded as part of the electronic supplementary material.

## References

[1] Konstantopoulos K, Kukreti S, McIntire LV 1998 Biomechanics of cell interactions in shear fields. Adv. Drug Deliv. Rev. 33, 141–164. (doi:10.1016/S0169-409X(98)00024-6) 10837657 10.1016/s0169-409x(98)00024-6

[2] McClatchey PM, Hannen E, Thomas S 2016 Microfluidic platforms for the interrogation of intravascular cellular trafficking mechanisms influenced by hemodynamic forces. In Microscale technologies for cell engineering (eds Singh A, Gaharwar AA), pp. 197–218. Berlin, Germany: Springer International Publishing.

[3] McEver R 1997 Selectin-carbohydrate interactions during inflammation and metastasis. Glycoconj. J. 14, 585–591. (doi:10.1023/A:1018584425879) 9298691 10.1023/a:1018584425879

[4] Simon SI, Green CC 2005 Molecular mechanics and dynamics of leukocyte recruitment during inflammation. Annu. Rev. Biomed. Eng. 7, 151–185. (doi:10.1146/annurev.bioeng.7.060804.100423) 16004569 10.1146/annurev.bioeng.7.060804.100423

[5] Somers WS, Tang J, Shaw GD, Camphausen RR 2000 Insights into the molecular basis of leukocyte tethering and rolling revealed by structures of P- and E-selectin bound to SLeX and PSGL-1. Cell 103, 467–479. (doi:10.1016/S0092-8674(00)00138-0) 11081633 10.1016/s0092-8674(00)00138-0

[6] Hanley WD, Napier SL, Burdick MM, Schnaar RL, Sackstein R, Konstantopoulos K 2006 Variant isoforms of CD44 are P- and L-selectin ligands on colon carcinoma cells. FASEB J. 20, 337–339. (doi:10.1096/fj.05-4574fje) 16352650 10.1096/fj.05-4574fje

[7] Napier SL, Healy ZR, Schnaar RL, Konstantopoulos K 2007 Selectin ligand expression regulates the initial vascular interactions of colon carcinoma cells: the roles of CD44v and alternative sialofucosylated selectin ligands. J. Biol. Chem. 282, 3433–3441. (doi:10.1074/jbc.M607219200) 17135256 10.1074/jbc.M607219200

[8] Thomas SN, Zhu F, Schnaar RL, Alves CS, Konstantopoulos K 2008 Carcinoembryonic antigen and CD44 variant isoforms cooperate to mediate colon carcinoma cell adhesion to E- and L-selectin in shear flow. J. Biol. Chem. 283, 15 647–15 655. (doi:10.1074/jbc.M800543200) 10.1074/jbc.M800543200 PMC2414264 18375392

[9] Thomas SN, Schnaar RL, Konstantopoulos K 2009 Podocalyxin-like protein is an E-/L-selectin ligand on colon carcinoma cells: comparative biochemical properties of selectin ligands in host and tumor cells. Am. J. Physiol. Cell Physiol. 296, C505–C513. (doi:10.1152/ajpcell.00472.2008) 19118161 10.1152/ajpcell.00472.2008 PMC2660269

[10] Alves CS, Burdick MM, Thomas SN, Pawar P, Konstantopoulos K 2008 The dual role of CD44 as a functional P-selectin ligand and fibrin receptor in colon carcinoma cell adhesion. Am. J. Physiol. Cell Physiol. 294, C907–C916. (doi:10.1152/ajpcell.00463.2007) 18234849 10.1152/ajpcell.00463.2007

[11] Aigner S, Ramos CL, Hafezi-Moghadam A, Lawrence MB, Friederichs J, Altevogt P, Ley K 1998 CD24 mediates rolling of breast carcinoma cells on P-selectin. FASEB J. 12, 1241–1251. 9737727 10.1096/fasebj.12.12.1241

[12] Dallas MR, Chen SH, Streppel MM, Sharma S, Maitra A, Konstantopoulos K 2012 Sialofucosylated podocalyxin is a functional E- and L-selectin ligand expressed by metastatic pancreatic cancer cells. Am. J. Physiol. Cell Physiol. 303, C616–C624. (doi:10.1152/ajpcell.00149.2012) 22814396 10.1152/ajpcell.00149.2012 PMC3468350

[13] Zhu C, Yago T, Lou J, Zarnitsyna VI, McEver RR 2008 Mechanisms for flow-enhanced cell adhesion. Ann. Biomed. Eng. 36, 604–621. (doi:10.1007/s10439-008-9464-5) 18299992 10.1007/s10439-008-9464-5 PMC2633097

[14] King MR, Hammer DD 2001 Multiparticle adhesive dynamics: hydrodynamic recruitment of rolling leukocytes. Proc. Natl Acad. Sci. USA 98, 14 919–14 924. (doi:10.1073/pnas.261272498) 11752440 10.1073/pnas.261272498 PMC64959

[15] Yago T, Zarnitsyna VI, Klopocki AG, McEver RP, Zhu C 2007 Transport governs flow-enhanced cell tethering through L-selectin at threshold shear. Biophys. J. 92, 330–342. (doi:10.1529/biophysj.106.090969) 17028146 10.1529/biophysj.106.090969 PMC1697837

[16] Dong C, Lei XX 2000 Biomechanics of cell rolling: shear flow, cell-surface adhesion, and cell deformability. J. Biomech. 33, 35–43. (doi:10.1016/S0021-9290(99)00174-8) 10609516 10.1016/s0021-9290(99)00174-8

[17] Chang KC, Hammer DD 1999 The forward rate of binding of surface-tethered reactants: effect of relative motion between two surfaces. Biophys. J. 76, 1280–1292. (doi:10.1016/S0006-3495(99)77291-7) 10049312 10.1016/S0006-3495(99)77291-7 PMC1300108

[18] Yago T, Wu J, Wey CD, Klopocki AG, Zhu C, McEver RR 2004 Catch bonds govern adhesion through L-selectin at threshold shear. J. Cell Biol. 166, 913–923. (doi:10.1083/jcb.200403144) 15364963 10.1083/jcb.200403144 PMC2172126

[19] Constantin G, Majeed M, Giagulli C, Piccio L, Kim JY, Butcher EC, Laudanna C 2000 Chemokines trigger immediate β2 integrin affinity and mobility changes: differential regulation and roles in lymphocyte arrest under flow. Immunity 13, 759–769. (doi:10.1016/S1074-7613(00)00074-1) 11163192 10.1016/s1074-7613(00)00074-1

[20] Atarashi K, Hirata T, Matsumoto M, Kanemitsu N, Miyasaka M 2005 Rolling of Th1 cells via P-selectin glycoprotein ligand-1 stimulates LFA-1-mediated cell binding to ICAM-1. J. Immunol. 174, 1424–1432. (doi:10.4049/jimmunol.174.3.1424) 15661900 10.4049/jimmunol.174.3.1424

[21] Kuwano Y, Spelten O, Zhang H, Ley K, Zarbock A 2010 Rolling on E- or P-selectin induces the extended but not high-affinity conformation of LFA-1 in neutrophils. Blood 116, 617–624. (doi:10.1182/blood-2010-01-266122) 20445017 10.1182/blood-2010-01-266122 PMC3324292

[22] Alon R, Ley K 2008 Cells on the run: shear-regulated integrin activation in leukocyte rolling and arrest on endothelial cells. Curr. Opin. Cell Biol. 20, 525–532. (doi:10.1016/j.ceb.2008.04.003) 18499427 10.1016/j.ceb.2008.04.003 PMC5912339

[23] Alon R, Dustin MM 2007 Force as a facilitator of integrin conformational changes during leukocyte arrest on blood vessels and antigen-presenting cells. Immunity 26, 17–27. (doi:10.1016/j.immuni.2007.01.002) 17241958 10.1016/j.immuni.2007.01.002

[24] Goldman AJ, Cox RG, Brenner H 1967 Slow viscous motion of a sphere parallel to a plane wall—II Couette flow. Chem. Eng. Sci. 22, 653–660. (doi:10.1016/0009-2509(67)80048-4)

[25] Donea J, Huerta A, Ponthot JP, Rodríguez-Ferran A 2004 Arbitrary Lagrangian–Eulerian methods. In Encyclopedia of computational mechanics. Hoboken, NJ: John Wiley & Sons, Ltd.

[26] Oh J, Edwards EE, McClatchey PM, Thomas SS 2015 Analytical cell adhesion chromatography reveals impaired persistence of metastatic cell rolling adhesion to P-selectin. J. Cell Sci. 128, 3731–3743. (10.1242/jcs.166439) 26349809 10.1242/jcs.166439 PMC4631778

[27] Tang JL, Schoenwald K, Potter D, White D, Sulchek T 2012 Bifunctional Janus microparticles with spatially segregated proteins. Langmuir 28, 10 033–10 039. (doi:10.1021/la3010079) 22624704 10.1021/la3010079 PMC3428262

[28] Tissot O, Pierres A, Foa C, Delaage M, Bongrand P 1992 Motion of cells sedimenting on a solid surface in a laminar shear flow. Biophys. J. 61, 204–215. (doi:10.1016/S0006-3495(92)81827-1) 1540690 10.1016/S0006-3495(92)81827-1 PMC1260234

[29] Ramos CL, Huo Y, Jung U, Ghosh S, Manka DR, Sarembock IJ, Ley K 1999 Direct demonstration of P-selectin- and VCAM-1-dependent mononuclear cell rolling in early atherosclerotic lesions of apolipoprotein E-deficient mice. Circ. Res. 84, 1237–1244. (doi:10.1161/01.RES.84.11.1237) 10364560 10.1161/01.res.84.11.1237

[30] Lawrence MB, Kansas GS, Kunkel EJ, Ley K 1997 Threshold levels of fluid shear promote leukocyte adhesion through selectins (CD62 L,P,E). J. Cell Biol. 136, 717–727. (doi:10.1083/jcb.136.3.717) 9024700 10.1083/jcb.136.3.717 PMC2134292

[31] Korn CB, Schwarz UU 2008 Dynamic states of cells adhering in shear flow: from slipping to rolling. Phys. Rev. E 77, 041904 (doi:10.1103/PhysRevE.77.041904) 10.1103/PhysRevE.77.041904 18517653

[32] Schmidtke DW, Diamond SS 2000 Direct observation of membrane tethers formed during neutrophil attachment to platelets or P-selectin under physiological flow. J. Cell Biol. 149, 719–730. (doi:10.1083/jcb.149.3.719) 10791984 10.1083/jcb.149.3.719 PMC2174847

[33] Chang KC, Tees DF, Hammer DD 2000 The state diagram for cell adhesion under flow: leukocyte rolling and firm adhesion. Proc. Natl Acad. Sci. USA 97, 11 262–11 267. (doi:10.1073/pnas.200240897) 10.1073/pnas.200240897 PMC17188 11005837

[34] Gallant ND, Michael KE, Garcia AA 2005 Cell adhesion strengthening: contributions of adhesive area, integrin binding, and focal adhesion assembly. Mol. Biol. Cell 16, 4329–4340. (10.1091/mbc.E05-02-0170) 16000373 10.1091/mbc.E05-02-0170 PMC1196341

[35] Park EY, Smith MJ, Stropp ES, Snapp KR, DiVietro JA, Walker WF, Schmidtke DW, Diamond SL, Lawrence MM 2002 Comparison of PSGL-1 microbead and neutrophil rolling: microvillus elongation stabilizes P-selectin bond clusters. Biophys. J. 82, 1835–1847. (doi:10.1016/S0006-3495(02)75534-3) 11916843 10.1016/S0006-3495(02)75534-3 PMC1301981

[36] Smith MJ, Berg EL, Lawrence MM 1999 A direct comparison of selectin-mediated transient, adhesive events using high temporal resolution. Biophys. J. 77, 3371–3383. (doi:10.1016/S0006-3495(99)77169-9) 10585960 10.1016/S0006-3495(99)77169-9 PMC1300609

[37] Chen S, Springer TT 2001 Selectin receptor--ligand bonds: formation limited by shear rate and dissociation governed by the Bell model. Proc. Natl Acad. Sci. USA 98, 950–955. (doi:10.1073/pnas.98.3.950) 11158576 10.1073/pnas.98.3.950 PMC14690

[38] Alon R, Hammer DA, Springer TT 1995 Lifetime of the P-selectin-carbohydrate bond and its response to tensile force in hydrodynamic flow. Nature 374, 539–542. (doi:10.1038/374539a0) 7535385 10.1038/374539a0

[39] Sun C, Migliorini C, Munn LL 2003 Red blood cells initiate leukocyte rolling in postcapillary expansions: a lattice Boltzmann analysis. Biophys. J. 85, 208–222. (doi:10.1016/S0006-3495(03)74467-1) 12829477 10.1016/S0006-3495(03)74467-1 PMC1303078

[40] Mao W, Alexeev A 2014 Motion of spheroid particles in shear flow with inertia. J. Fluid Mech. 749, 145–166. (doi:10.1017/jfm.2014.224)

[41] Kilimnik A, Mao W, Alexeev A 2011 Inertial migration of deformable capsules in channel flow. Phys. Fluids 23, 123302 (doi:10.1063/1.3664402)

[42] Noguchi H, Gompper G 2005 Shape transitions of fluid vesicles and red blood cells in capillary flows. Proc. Natl Acad. Sci. USA 102, 14 159–14 164. (doi:10.1073/pnas.0504243102) 10.1073/pnas.0504243102 PMC1242298 16186506

[43] Fedosov DA, Caswell B, Karniadakis GG 2010 A multiscale red blood cell model with accurate mechanics, rheology, and dynamics. Biophys. J. 98, 2215–2225. (doi:10.1016/j.bpj.2010.02.002) 20483330 10.1016/j.bpj.2010.02.002 PMC2872218

